# Supplementary material for: Engineering microscale systems for fully autonomous intracellular neural interfaces
Source: Microsyst Nanoeng. 2020 Feb 10;6:1. doi: 10.1038/s41378-019-0121-y (PMC8433365; doi:10.1038/s41378-019-0121-y)
Supplement: Supplementary file 1 — Supplementary Figure 1 [file 41378_2019_121_MOESM1_ESM.pdf]

A

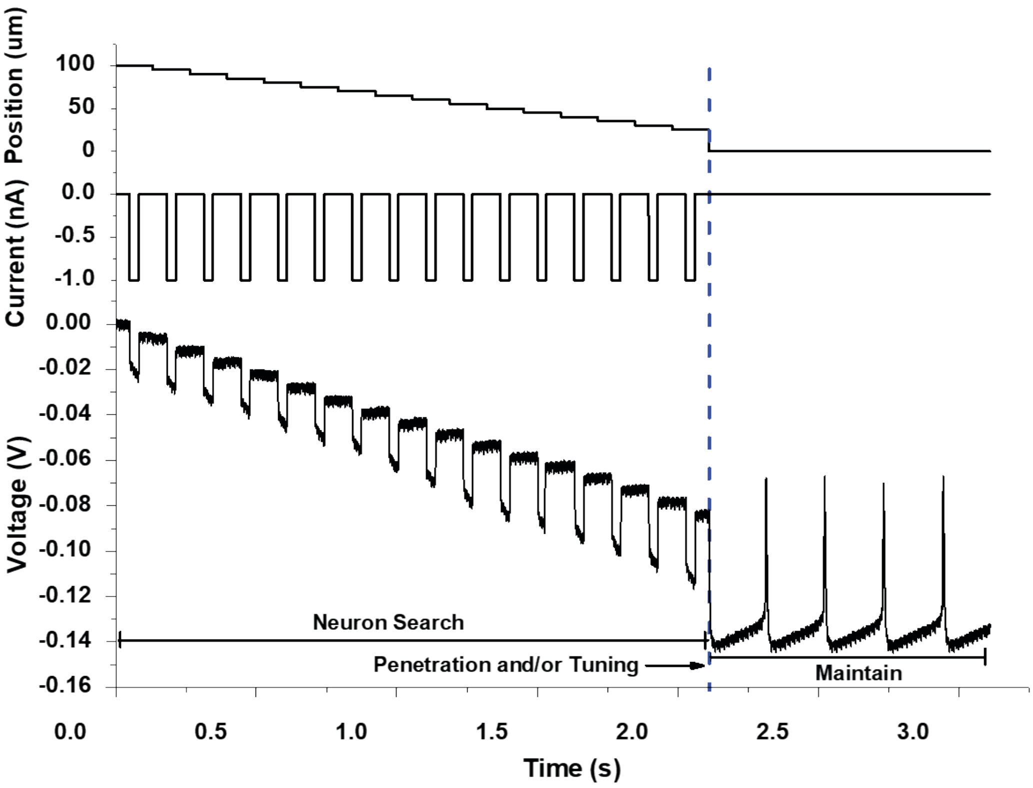


B

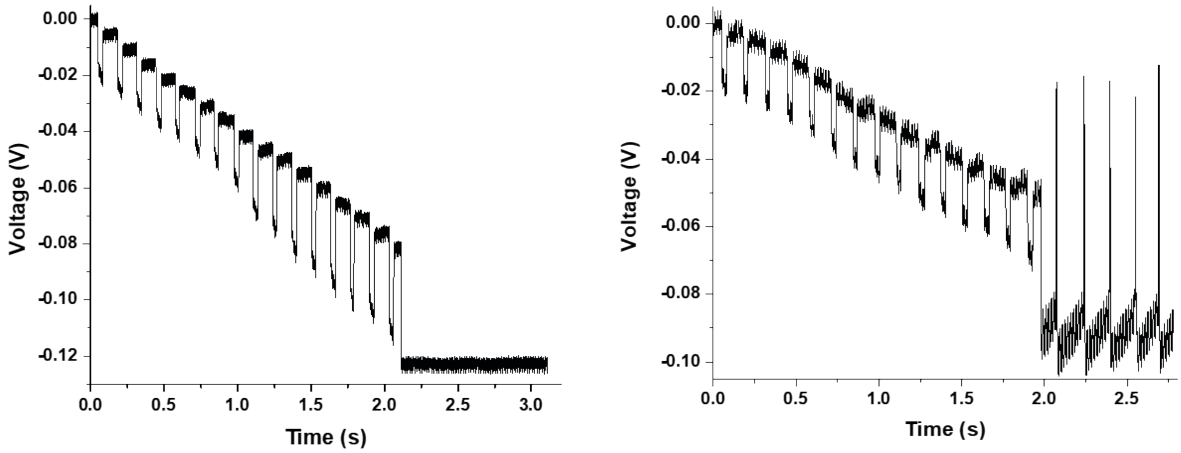


**Supplementary Fig.1:** Validation of the closed-loop control after integration of controller with a GP microelectrode and a conventional hydraulic microdrive. (A) Illustration of a typical trial of the closed loop control - the controller moved the electrode using the microdrive in steps of 5 µm towards a neuron in the isolated abdominal ganglion, until the electrical impedance of the tip was above the threshold value of 8 MΩ, beyond which the controller switched to the 'Penetration and/or Tuning' mode to impale the neuron and obtained intracellular recordings. (B) Representative plots showing performance of the controller in 2 additional neurons with good quality resting potentials (RP) and/or action potentials (AP).
